# On-Substrate Preparation of a Poly(triphenylamino azomethine) for Electrochromic Devices

**DOI:** 10.3390/polym16172440

**Published:** 2024-08-28

**Authors:** Heather L. Filiatrault, Kacper Muras, Monika Wałęsa-Chorab, W. G. Skene

**Affiliations:** 1 Laboratoire de Caractérisation Photophysique des Matériaux Conjugués, Département de Chimie, Université de Montréal, Montreal, QC H3C 3J7, Canada; 2 Faculty of Chemistry, Adam Mickiewicz University in Poznań, 61-614 Poznań, Poland; 3 Institut Courtois, Université de Montréal, Montreal, QC H3C 3J7, Canada

**Keywords:** polyazomethine, on-substrate polymerization, large-area electrochromic device

## Abstract

An electroactive polyazomethine was prepared directly on a transparent electrode by the polycondensation of bis(triphenylamine) dialdehyde and its complementary methoxytriphenylamine diamine. The *spray-and-bake* method of coating the electrode for preparing electrochromic layers could be upscaled to prepare working devices larger than standard test devices. The film prepared by thermally annealing the complementary monomers was both electroactive and switched its color with an applied potential. The yellow electrochromic polyazomethine could be electrochemically oxidized reversibly to obtain a blue film. The electrochromic test device fabricated from the polyazomethine was operated upwards of 1 h for performance assessment. The electrochromic response times of the electrochromic device were ca. 3.3 and 1.2 s for the coloration and bleaching, respectively. The upscaled device prepared by the straightforward coating approach had consistent metrics with the small-area test device.

## 1. Introduction

Conjugated polymers that can reversibly change their color with an applied potential are beneficial for use as the active layer in operating electrochromic devices [1,2,3]. This is because their color can be adjusted contingent to the molecular structure of their constitutional components [4]. Polymers that are soluble in common solvents have an additional benefit in terms of their processability for use as electrochromes. For example, they can be deposited as homogeneous layers on substrates of various sizes using large-area coating methods. Electrochromic devices of various sizes can therefore be fabricated and extended to include flexible substrates. Given the applied potential required to drive the color change process with organic polymers occurs at relatively low voltages requiring low power consumption, organic electrochromic devices are considered as sustainable devices [5,6,7].

To take advantage of the processing and subsequent material property virtues of conjugated polymers, they must be soluble in a given solvent. The solubility must be rationally designed into the polymer at the monomer level. This requires incorporating either branched or long alkyl pendant chains into the functional unit prior to polymerization [8,9]. These additional synthetic steps increase the quantity of reagents and solvents required to prepare the electrochromic material. This ultimately offsets the sustainable benefits of the electrochromic device. Polymer solubility is also a challenge during device fabrication. This is because the deposited layer can delaminate from the surface and mix with layers that are deposited on top. The layer mixing can result in inhomogeneous coloration and reduced performance of the operating device [10].

The number of synthetic steps required to prepare reactive monomers and their processing challenges can be addressed with insoluble electrochromic polymers. Rather than employing soluble polymers, solution processable monomer can be polymerized directly on the device electrode. This has the advantage of requiring fewer synthetic steps. Moreover, solubility groups are not required to be integrated in the monomer. Indeed, monomers with a diverse range of functionality, such as oxetanes and vinylenes to name but a few, have been used to make films that are robustly immobilized on the device electrode by their in situ polymerization [11,12,13,14]. The polymerization directly on the substrates gives rise to desired films that do not delaminate. However, the degree of conjugation of the resulting polymer is often limited compared to their soluble polymer counterpart. This affects the electrochromic properties.

Various deposition methods have been be used to prepare thin film coatings of conjugated polymers on electrodes [15,16,17]. Vapor phase polymerization for deposition coatings has garnered widespread acceptance. This is owing to its straightforward approach that uniquely involves evaporating the monomers under reduced pressure. The vaporized monomers spontaneously polymerizes when it condenses on the surface [18,19]. Vapor phase polymerization has the advantage of not requiring a solvent. Indeed, the monomers are not required to be soluble in the solvent. This is offset by a range of constraints that limit adopting the process for preparing films on a commercial level. For example, the size of surfaces that can be coated are limited by vacuum dimensions that can result in prohibitive production costs for large area substrates for industrial use [20]. As such, the polymerization method is often restricted to the laboratory setting. The range of polymers that can be prepared by vapor phase polymerization is further limited by their vapor pressure; lower pressures are required to vaporize less volatile monomers [21]. This precludes polymerizing monomers with high molecular weight. Another limitation is the thermal stability of the monomers, they must be stable at the vaporization temperature. Moreover, copolymers are challenging to achieve owing to the often different vapor pressures of the comonomers.

Electrochemical polymerization has also exceled for preparing insoluble thin films of conjugated polymers from soluble monomers. Here, the radical ion of the given monomer is generated by applying the required potential. A thin film of the polymer is subsequently adsorbed directly on the working electrode by step-growth polymerization via the self-reaction of the radical ions [22,23]. Electropolymerization has the advantage of fast kinetics [24] with the electroactive polymer being formed in a short period of time with high adhesion [25]. The thickness of the deposited layer can in part be controlled by adjusting the charge applied to the electrode during electropolymerization [26,27]. Advantages of electropolymerization are initiators, UV light, and heating not being required. In contrast, high concentrations of the monomer are required. The monomer must therefore be highly soluble in one of the limited number of organic solvents that are electrochemically conductive with an electrolyte. Moreover, the monomer must be electroactive. Generating the required radical ion must occur within the electrochemical operating limits of the solvent. While electrochemical polymerization is ideal for preparing homopolymers, preparing copolymers is challenging. This is because the copolymerization requires similar electrochemical activity for the monomers to be polymerized. Another limitation of electrochemical polymerization is the area of the coating that can be prepared, being limited to small size test surfaces [28].

An appropriate method for fabricating large-scale patterned images of conjugated polymers on substrates is inkjet printing [29,30]. This method is also versatile for coating films on diverse substrates including rigid, bendable, and stretchable surfaces [31]. Inkjet printing is not without its limitations. For example, the requirement of inks is they must be of an appropriate viscosity [32]. This requires a good solubility of the polymers to be coated in the ink solvent. Viscosity modifiers are also necessary to achieve the required viscosity. A noncontiguous film is also produced by the coffee stain effect where the ink is not optimized [33]. This ring-like shape occurs when the particles of the nonvolatile solute in the ink accumulate at the edge of the drop.

Spray coating in part overcomes the limitations of other deposition methods. The true virtue of this deposition method is its versatility to coat surfaces of various scales [34]. Indeed, coatings ranging from laboratory size to commercial applications are possible [35]. Moreover, the coating thickness can be adjusted according to the number of passes sprayed [36]. The quality of the films can also be controlled by both the speed and the pattern sprayed. While the limitation of the polymer solubility in the delivery solvent holds true for spray coating, this can be overcome by using soluble monomers rather than polymers. Desired conjugated polymers can therefore be prepared by the in situ thermal polymerization of monomers coated by spray coating [13,37,38].

Azomethines are viable candidates for conjugated films that can be directly prepared on the electrode via spray-coating. This is in part owing to their straightforward preparation by the condensation of complementary dialdehydes and diamines on a surface with a given catalyst [10,39,40]. This is further promoted by the solubility of the complementary monomers in solvents that are compatible for spray coating. A benefit of azomethines is the resulting heteroatomic bond has an increased degree of conjugation compared to its individual constitutional components [41]. As such, colors spanning the visible region similar to conjugated polymers are possible by the judicious choice of complementary amines and aldehydes [42,43]. Azomethines have proven to be ideal candidates for active layers in devices in part because of their air stability and good thermal resistance [44]. Indeed, azomethines have been used in plastic electronic devices including photovoltaics [45,46], memory devices [47], or organic light-emitting diodes [48,49]. They also can be used as active materials in electrochemical sensors [50] and as electrochromic materials [12,51].

Leveraging the known insolubility of polyazomethines [52,53], we herein present an electrochromic polyazomethine that is immobilized on the electrode. Our approach entails the thermal annealing of complementary amine/dialdehyde monomers directly on the surface after their deposition by spray coating [10,39,40]. By judiciously choosing monomers with electrochromic properties such as electrochemical reversibility and color change with applied potential, we demonstrate the *spray-and-bake* approach can be used for preparing active layers that enable electrochromic devices. The universality of the facile film forming method for upscaling the fabrication of functioning electrochromic devices beyond common test-size devices is also demonstrated.

## 2. Experimental Procedure

### 2.1. General Procedures

All chemicals and consumables were obtained from commercial sources (Sigma-Aldrich, Oakville, ON, Canada) and they were used as received unless otherwise stated. Anhydrous solvents were obtained from an activated alumina column solvent purification system.

### 2.2. Spectroscopic and Spectroelectrochemical Measurements

Absorption spectra were measured with a Varian Cary 500 spectrophotometer. Spectroelectrochemistry in solution was performed with TBAPF_6_ (0.1 M) as the electrolyte solutions in anhydrous dichloromethane. An optically narrow cuvette (1.7 mm) with a gold honeycomb working (Pine Research) was coupled to the spectrophotometer and the potentiostat. A silver wire was used as the pseudo-reference electrode.

### 2.3. Electrochemical Measurements

Electrochemical measurements were performed with a BioLogic VSP potentiostat. Compounds (1 mM) were dissolved in anhydrous and deaerated dichloromethane along with the electrolyte. A platinum button electrode was used as the working electrode while a platinum wire and a saturated Ag/AgCl electrode were used as the auxiliary and the reference electrodes, respectively. Equimolar ferrocene was added as an internal standard (E^o′^ = 460 mV vs. SCE) [54] to calibrate the potentials at the end of measurements relative to the reversible Fc/Fc^+^ couple. ITO coated glass slides (Delta Technologies Ltd.) were used for evaluating the polymerized films.

### 2.4. On-Substrate Polymerization

Dialdehyde **A** (2 mg/mL) and diamine **B** (1.12 mg/mL) were dissolved in dichloromethane. The homogeneous solution was spray coated on pre-cleaned ITO glass substrates (2.5 cm × 2.5 cm) with an automatic spray coater (Ultrasonic Sonic Systems, Prism-Ultra-Coat) at 1.5 mL/min, 10 psi, and a spray height of 100 mm. After depositing the monomers, the ITO substrates were heated at 110 °C for 1 h in a saturated trifluoroacetic acid (TFA) atmosphere. **CAUTION**: the acid is corrosive, and it should be handled with care and the processing of the films with acid vapors should be performed in a fume hood. The polymerized films were rinsed successively with a triethylamine solution in dichloromethane and followed by neat dichloromethane. Afterwards, they were dried under a stream of air.

### 2.5. Electrochromic Device

**Figure 1:**
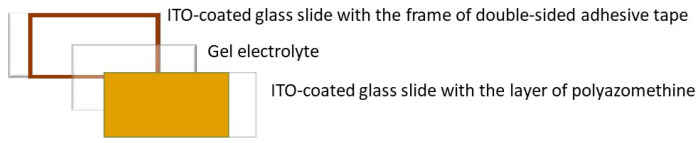
Schematic representation of the simple sandwich electrochromic device.

Transmissive electrochromic devices were fabricated according to previously described methods [55]. In short, a two-layer sandwich device was fabricated consisting of the electrochrome on-substrate prepared layer and the gel electrolyte as is schematically depicted in Figure 1.

First, a frame of double-sided adhesive tape was made on an ITO-coated glass slide. Then, the gel electrolyte was spread inside the frame and the second ITO-glass coated with the layer of polyazomethine was placed on the top of the gel layer. The electrochemical properties of the device were measured with a potentiostat and the spectroscopic properties were measured with the stated spectrometer.

### 2.6. Synthesis

*N*^4^,*N*^4^,*N*^4′^,*N*^4′^-tetraphenylbiphenyl-4,4′-diamine (**1**) [13]. Prepared identically to the reported method (1.23 g, 85%). ^1^H NMR (400 MHz, CDCl_3_) δ 7.47 (d, J = 8.7 Hz, 4H), 7.29 (dd, J = 8.5, 7.4 Hz, 8H), 7.19–7.11 (m, 12H), 7.05 (t, J = 7.3 Hz, 4H) ppm. ^13^C NMR (100 MHz, CDCl_3_) δ 147.9, 146.9, 134.9, 129.4, 127.4, 124.4, 124.2, 122.9 ppm. HR-MS m/z = calcd. 489.2325; found 489.2325 (M+H)^+^. Anal. Calcd for C_36_H_28_N_2_ (488.22): C, 88.49; H, 5.78; N, 5.73. Found: C, 88.46; H, 5.80; N, 5.72.

4,4′-(Biphenyl-4,4′-diylbis(phenylazanediyl))dibenzaldehyde **A** [13]. Prepared identically to the reported method (0.65 g, 81%). ^1^H NMR (400 MHz, CDCl_3_) δ 9.83 (s, 2H), 7.71 (d, J = 8.9 Hz, 4H), 7.55 (d, J = 8.8 Hz, 4H), 7.40–7.32 (m, 4H), 7.26–7.16 (m, 10H), 7.08 (d, J = 8.7 Hz, 4H) ppm. ^13^C NMR (100 MHz, CDCl_3_) δ 190.6, 153.3, 146.2, 145.6, 136.8, 131.5, 130.0, 129.5, 128.1, 126.5, 126.3, 125.4, 119.9 ppm. HR-MS calcd. 545.2224, found 545.2207 (M+H)^+^. Anal. Calcd for C_38_H_28_N_2_O_2_ (544.21): C, 83.80; H, 5.18; N, 5.14. Found: C, 83.81; H, 5.17; N, 5.15.

4,4′-Dinitro-4″-methoxytriphenylamine (**2**) [56,57]. To a solution of *p*-anisidine (1.0 g, 8.1 mmol) in DMSO (7 mL) *p*-fluoronitrobenzene (2.3 g, 16.3 mmol) and cesium fluoride (2.5 g, 16.5 mmol) were added. The mixture was then heated at 120 °C for 24 h. Afterwards, the mixture was poured into cold methanol (100 mL) and the orange-red solid was filtered and dried. The crude product was purified by column chromatography (SiO_2_, dichloromethane:hexane 9:1) to afford a solid (2.27 g, 77%). ^1^H NMR (400 MHz, CDCl_3_) δ 8.30 (d, *J* = 9.1 Hz, 4H), 7.55 (d, *J* = 8.7 Hz, 2H), 7.15 (d, *J* = 9.2 Hz, 4H), 7.05 (d, *J* = 8.7 Hz, 2H), 3.43 (s, 3H) ppm. ^13^C NMR (100 MHz, CDCl_3_) δ 154.3, 141.4, 140.5, 126.8, 125.3, 123.8, 116.1, 114.4, 55.6 ppm. HRMS *m*/*z* = calcd 366.3469; found 336.3463 (M+H)^+^. Anal. Calcd for C_19_H_15_N_3_O_5_ (365.10): C, 62.46; H, 4.14; N, 11.50. Found: C, 62.44; H, 4.17; N, 11.48.

4,4′-Diamino-4″-methoxytriphenylamine (**B**) [56,57]. To a solution of **2** (1.5 g, 4.1 mmol) in methanol (10 mL) Pd/C (10% mol) was added. Hydrazine hydrate (1.3 mL) was then added dropwise to the suspension. The mixture was heated at 100 °C for 10 h. The hot mixture was then filtered through celite and it was then cooled to obtain white needles. The product was collected by filtration and dried (0.96 g, 77%). ^1^H NMR (400 MHz, CDCl_3_) δ 6.93 (d, *J* = 8.5 Hz, 2H), 6.87 (d, *J* = 8.1 Hz, 4H), 6.77–6.73 (m, 2H), 6.59 (d, *J* = 8.6 Hz, 4H), 3.77 (s, 3H), 3.46 (s, 4H). ^13^C NMR (100 MHz, CDCl_3_) δ 154.4, 151.4, 141.4, 125.4, 123.8, 117.1, 116.2, 114.5, 55.7 ppm. HRMS *m*/*z* = calcd. 306.1601; found 306.1603 (M+H)^+^. Anal. Calcd for C_19_H_19_N_3_O (305.15): C, 74.73; H, 6.27; N, 13.76. Found: C, 74.71; H, 6.29; N, 13.74.

## 3. Results and Discussion

### 3.1. Polyazomethine Preparation

**Figure 2:**
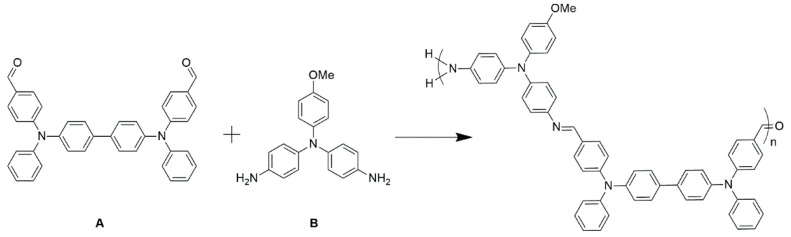
Polymerization scheme for the preparation of the polyazomethine by polycondensation of monomers **A** and **B**.

The monomer **A** was chosen because the electrochemical reversibility of the bistriphenylamine is comparable to its triphenylamine counterpart [58]. This was expected to enhance the switching performance of the final electrochromic device. The common element of both monomer **A** and **B** is the triphenylamine. This was chosen as a constitutional component of the resulting polyazomethine because it is electrochemically reversible. Moreover, it changes its color when oxidized. These are key properties desired for enabling electrochromic devices. The electrochromic polymer was prepared directly on the electrode. The requisite monomers were prepared according to known methods (Figure S1) [13,56,57]. A stock solution of an equimolar amount of dialdehyde **A** and diamine **B** in dichloromethane was spray-coated onto pre-cleaned ITO glass substrates. This solvent was chosen because it evaporates during spray coating. The monomers are therefore dry deposited on the substrate without runoff. This ensures a uniform and consistent film over the entire surface of the substrate. The resulting coated substrate was then annealed at 110 °C for 1 h (Figure 2). A saturated environment of TFA was used as the catalyst for the imine reaction. For this, a vial of TFA was placed beside the substrate and they were both covered with a Petri dish. At the annealing temperature, the acid evaporated. Sufficient vapor contacted the surface to catalyze azomethine formation. The annealing time also ensured diffusion of the catalyst throughout the film for its complete polymerization both laterally and medially. After polymerizing, the films were rinsed successively with a solution of triethylamine in dichloromethane and then neat dichloromethane. The base was chosen to neutralize the protonated amines, remove any acid embedded in the acid, and remove both any unreacted monomers and soluble oligomers from the polymer film.

**Figure 3:**
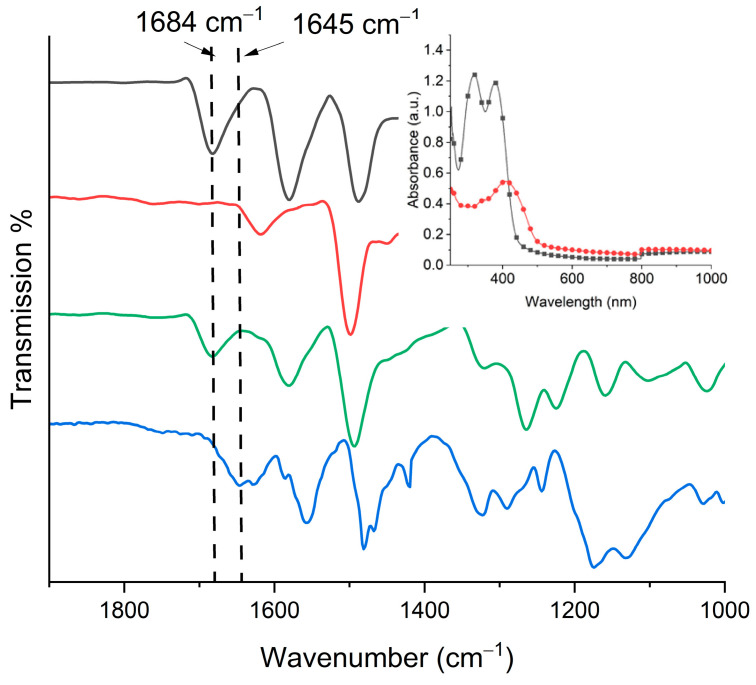
ATR FT-IR spectra of **A** (black), **B** (red), a thin film coating of equimolar **A** and **B** on ITO coated glass (green), and the polyazomethine film formed by thermally annealing followed by rinsing (blue). Inset: absorbance spectra of monomers **A** and **B** coated on the ITO glass substrate before polymerization (black) and the resulting polymerized film (red).

The thermal polymerization on the surface was confirmed by both FT-IR and absorption spectroscopy along with the persistence adhesion of the film to the substrate. This contrasts with the monomers and oligomers that are readily soluble and they are removed by rinsing the surface. As shown in Figure 3 (inset), the thin film of the monomers prior to their polymerization has two distinct absorptions. One peak absorbed at 316 nm while the other absorbed at 380 nm. After polymerization and rinsing the surface, the absorption of the film was bathochromically shifted to 412 nm. Visually, the transparent film becomes yellow with the *spray-and-bake* process. The color change is consistent with an increased degree of conjugation that arises upon azomethine formation [14]. This aside, the FT-IR data provide unequivocal evidence for polyazomethine formation of the film. This is according to the disappearance of the aldehyde peak at 1684 cm^−1^ for **A** concomitant with the formation of the N=CH stretching at 1645 cm^−1^ (Figure 3). Although the heteroatomic bond is a vibrationally disallowed transition, it nonetheless is apparent, albeit weakly.

**Figure 4:**
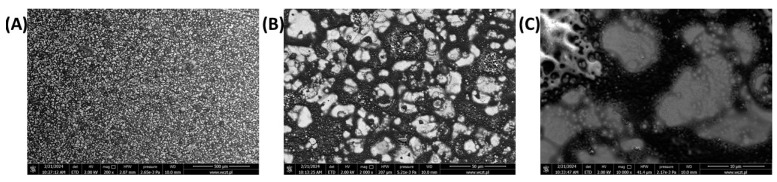
SEM images of the polyazomethine layer at magnifications 200× (**A**), 2000× (**B**) and 10,000× (**C**).

The surface morphology of the layer of the polyazomethine was investigated using scanning electron microscopy (SEM) (Figure 4). One can see that the surface of the ITO is entirely covered by a film of the polymer. The film is contiguous with no cracks. However, the surface is inhomogeneous and the film consists of aggregates. This is the result of the coating method. It is known that the quality of the annealed film correlates with the quality of the film deposited by spray-coating. This is underpinned by the user. While homogenous films of high quality can be obtained by spin-coating, spray coating is better suited for depositing on large areas.

### 3.2. Electrochemistry and Electrochromism

**Figure 5:**
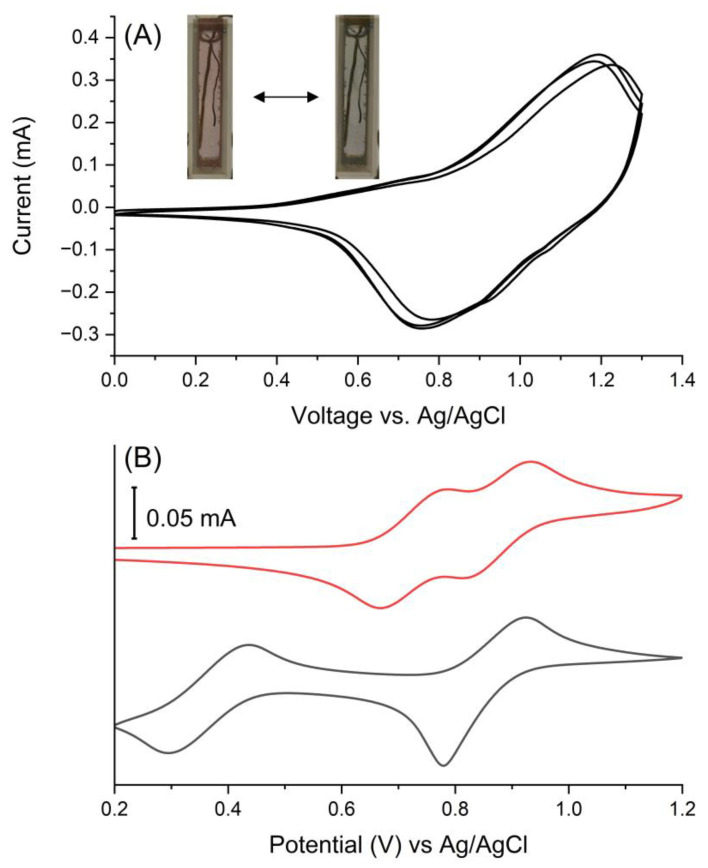
(**A**) Multiple cyclic voltammograms of the *spray-and-bake* polymerized azomethine on an ITO working electrode after rinsing with dichloromethane and measured in dichloromethane with TBAPF_6_ (0.1 M) as the supporting electrolyte. Inset: photographs of the polyazomethine film on ITO in the neutral (left) and oxidized (right) states. (**B**) Cyclic voltammograms of A (red) and B (black) measured in dichloromethane with TBAPF_6_ (0.1 M) as the supporting electrolyte with a Pt working electrode.

The electrochemical properties of the colored filmed were investigated by cyclic voltammetry. This was to confirm both its electroactivity and electrochromism. Of importance is the reversible redox behavior that is a key metric for electrochromic use. The targeted property was expected with the triphenylamines chosen for the polyazomethine. This is based on knowledge that arylamines undergo both reversible oxidation and color changes when oxidized [59,60]. Both monomers exhibited reversible oxidations (Figure 5B). The dialdehyde **A** underwent two reversible oxidations with anodic peak potentials (E_pa_) at +0.78 and +0.93 V. These were assigned to the stepwise oxidation of the biphenyl tetraphenyldiamine, forming the polaron and dipolaron [61]. Two reversible oxidations were also observed at E_pa_ +0.43 and +0.92 V for **B**. This step-wise oxidation confirms that the primary and the tertiary amines are electronically coupled [62]. Two oxidations were also observed for the polymer. One oxidation was irreversible at +0.80 V and the other at +1.45 V was reversible (Figure S3). Rinsing and immersing the polymer in fresh electrolyte solution afforded a unique reversible oxidation. The forward anodic peak potential occurred at +1.19 V and its associated reversible cathodic peak was at +0.75 V (Figure 5). The oxidation potential of the polymer was 260 mV more positive than its corresponding monomers. This is a result of the intrinsic electron withdrawing effect of the heteroatomic bond. The absence of the distinct oxidation in the monomers in the polymer cyclic voltammogram provides further evidence the spray-and-bake method yields the desired polyazomethine. The electrochromism of the film was confirmed by its change in color with applied potential. The color of the film changed from yellow to purple to blue for its neutral and oxidized states, respectively, while the yellow to purple color change was irreversible.

It is challenging to unequivocally assign the exact triphenylamine moiety that is oxidized in sequence upon applying a positive potential. Nonetheless, insight can be had from both chemical intuition and the electrochemical data of the constitutional monomers (Figure 5B). In short, applying a positive potential is expected to form a radical cation first on one of the arylamines of the bis(triphenylamine) region of the polymer. This is owing to its increased degree of conjugation that lowers the oxidation relative to other triphenylamine. Indeed, the oxidation occurring at the bis(triphenylamine) is supported by the cyclic voltammetric data of monomer **A** (Figure 5B). Courtesy of its conjugation, the other triarylamine of bis(triphenylamine) is oxidized nearly simultaneously within the film. The first oxidation causes the color to change from yellow to red. Increasing the applied positive potential next oxidized the unique triphenylamine that is conjugated with the azomethine. This is likely because of the electron withdrawing effect of the azomethine that increases the oxidation of the amine in comparison with both the bis(triphenylamine) and its corresponding constitutional component (Figure 5B). The color changes from red to blue with the second oxidation. The oxidations are accompanied with the migration of the perchlorate anions from the gel electrolyte to maintain the electrochemical neutrality. The proposed mechanism of the electrochromic response of the polyazomethine is summarized in Figure S4.

While the polyazomethine film was electrochromic, according to its change in color with applied potential, the performance observed in this half-device configuration was only qualitative. No information about the performance of the polyazomethine in an actual operating solid-state device can be derived from this configuration. As such, the suitability of the polyazomethine as the active color switching layer in a test-size operating electrochromic device was evaluated. The solid-state device was fabricated by sandwiching two ITO substrates together: one bearing a spray-coated polymerized film of polyazomethine prepared as described above and the other coated with an electrolyte gel. A double-sided tape spacer was used to isolate the two electrodes and adhere them together. The final device was assembled in an inert atmosphere. The assembled device was sealed with epoxy to prevent the diffusion of both moisture and oxygen into the layer during device operation.

**Figure 6:**
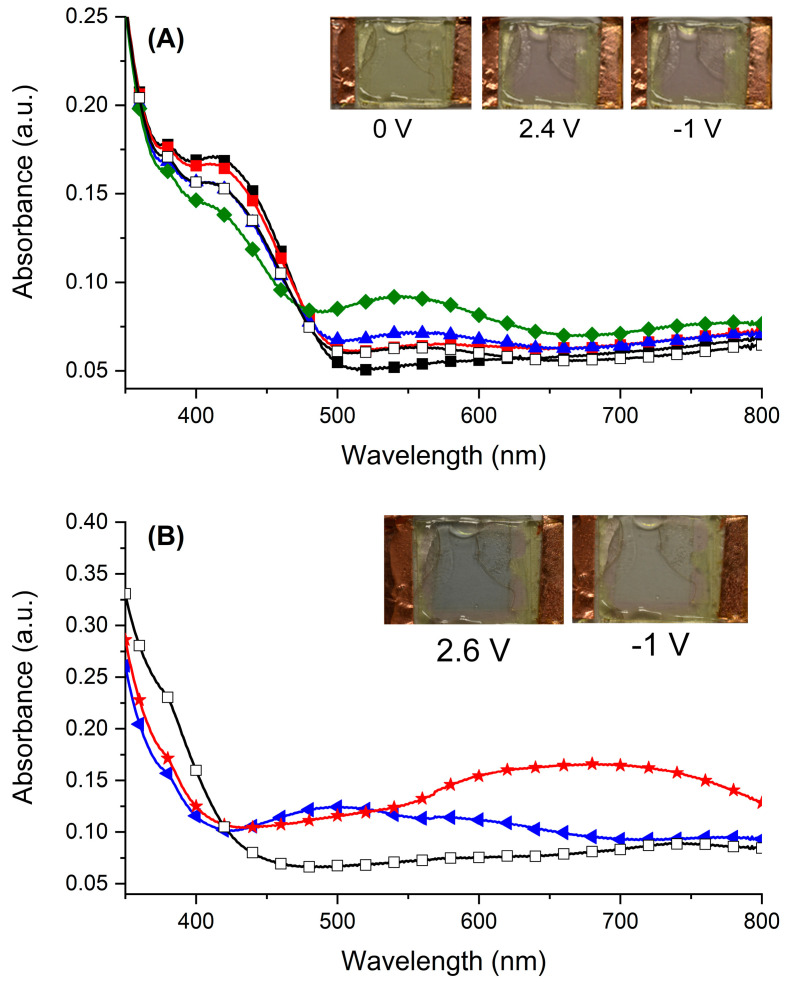
Absorbance spectra of the electrochromic test device at (**A**): 0 V (■), 2.1 V (■), 2.2 V (▲), 2.3 V (♦), −1.0 V (□). Inset: photographs of the electrochromic device at 0 (left), 2.4 (middle), and −1.0 (right) V. (**B**): 2.5 (◄), 2.6 (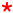), −1.0 V (□). Inset: photographs of the device at 2.6 (left), and −1.0 (right) V.

The electrochromism of the polyazomethine in the functioning device was investigated by measuring the change in absorption with the applied potential. The desired behavior of the electrochromic polymer film is obvious in Figure 6A. It is worthy to note that the applied potential in the device (+2.4 V) was greater than the oxidation potential that was observed by cyclic voltammetry. The larger potential required to induce the color was owing to the intrinsic resistance of the gel electrolyte. This aside, the absorption at 415 nm decreased with increasing the applied potential concomitant with the formation of a new peak centered at 545 nm. The electrochemically induced color change was from yellow to purple. This color change was pseudoreversible. The colored state was indeed bleached with an applied potential of −1 V, but it did not completely bleach. Also, the intensity of the colored state did not fully recover. When the oxidation potential was further increased to 2.6 V, the new absorption band having a maximum at 680 nm was formed and the perceived color changed from purple to blue (Figure 6B). The color change was reversible, and the color bleached with an applied potential of −1.0 V.

**Figure 7:**
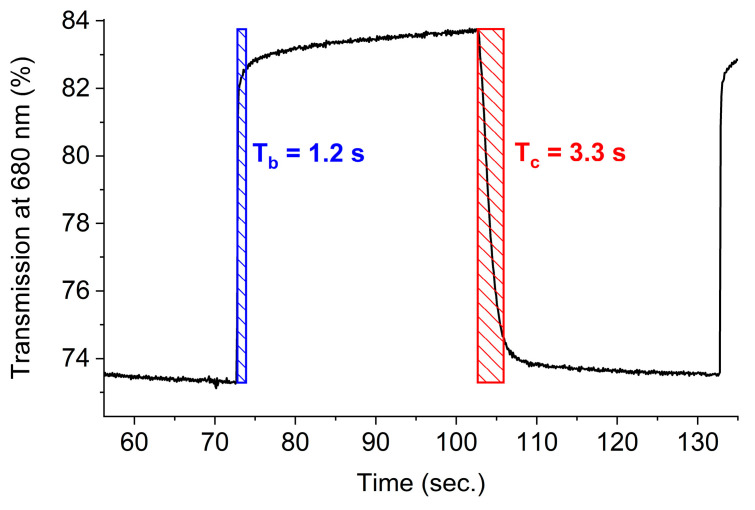
Coloration and bleaching times of the test-size electrochromic device derived from the change in percent transmission with applied potential.

The device was further investigated in terms of its electrochromic performance. This was performed by first investigating its color stability over many cycles of switching the potential. For this, the potential was switched between 2.3 V and −1.0 V at 30 s intervals while monitoring the change in transmission % for the oxidized state at 545 nm (Figure S5). An initial change in transmission of 8% was observed at 645 nm. This decreased to <2% after 1 h of cycling. This is not surprising as the color change was only partially reversible at these potentials. The reversible color change from purple to blue that was observed during switching of the potential between 2.6 and −1.0 V at 30 s intervals was more consistent (Figure S6). The initial change in transmission at 680 nm was 11% and it decreased to 5% after 1 h of cycling. The decrease in the difference between the colored and bleached states of the device was probably due to the polymer degrading during multiple oxidation/reduction cycles. This could also be caused by the overoxidation of the polymer [63] that results in its irreversible oxidation at the high potentials required to drive the device. The high applied potentials of 2.6 V and −1.0 V were required to induce the visible color changes with the device owing to the resistance of both the gel and electrochromic layers. While the polymer layer could potentially sustain multiple cycles of at lower potentials, reducing the applied potential would not improve the color contrast. An alternative solution would be to cross-link the film as increasing the number of polymer interconnects is known to improve chemical robustness [64,65]. This aside, the coloration (T_c,90_) and bleaching (T_b,90_) times were calculated according to the time required to reach a 90% change in transmission. The coloration and bleaching kinetics were 3.3 and 1.2 s, respectively (Figure 7).

The electrochromic performance of the on-substrate prepared polyazomethine was comparable to structurally similar other polyazomethines. This is evident from the compiled coloration and bleaching kinetics along with the perceived color changes summarized in Table 1. The color contrast of the on-substrate prepared polyazomethine was comparable to polyazomethines prepared from triphenylamine and either EDOT or thiophene [66]. In contrast, the color contrast derived of the polyazomethine prepared from triphenylamine and bisphenol was higher (85%) [67]. The coloration kinetics of the on-substrate polyazomethine were comparable to known triphenylamine polyazomethines [40,66,67,68]. For example, the coloration and bleaching times of a triphenylamine-tetraphenylethylene polyazomethine were 6.45 s and 1.07 s, respectively [68].

**Figure 8:**
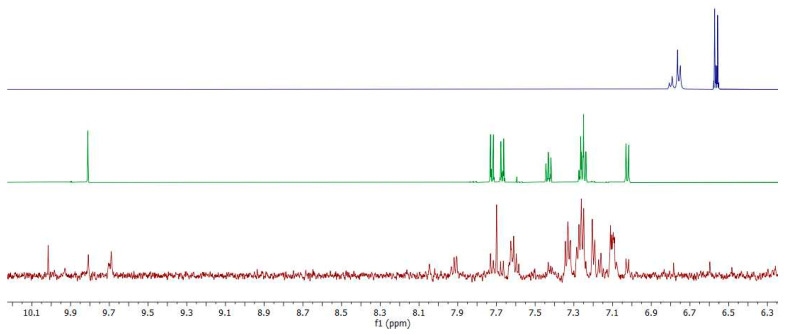
^1^H-NMR spectra of diamine **B** (blue), dialdehyde **A** (green) and the solution obtained after degradation of the polymer (red) in CD_3_CN.

The polymer film was subjected to multiple oxidation/reduction cycles in the half-device configuration. This consisted of the polymer film on the ITO electrode which was then immersed in an electrolyte solution (0.1 M TBAPF_6_) in acetonitrile. This configuration was used to investigate whether the loss of performance of the film during repeated redox cycles was from either its degradation or delamination of the electrochromic polymer from the surface. Delamination was expected from low molecule weight oligomers in the film whose solubility would be greater than its high molecular counterpart. The oligomers along with its potential hydrolysis to give the original monomers would readily be detected in the electrolyte solution by ^1^H-NMR spectroscopy. As such, the electrolyte solution was concentrated after the given number of redox cycles. Given the resonances of the electrolyte are outside the region of interest for measuring the oligomers and its corresponding monomers in the NMR spectrum, the electrolyte was not removed from the solid sample. The dialdehyde **A** is clearly present in the NMR spectrum (Figure 8) of the isolated delaminates. This is according to the characteristic CHO peak at 9.8 ppm (bottom panel) in comparison to the reference NMR spectrum of dialdehyde **A** (top panel). Additional aldehydes peaks in the 9.5 ppm–10.0 ppm region are also observed. These are from oligomers having various degrees of oligomerization, which are end-capped with the aldehyde monomer. This is supported by the characteristic azomethine signal at ca 8.0 ppm. Given monomer **A** was detected, its complementary monomer **B** should also be observed. This was not the case owing to its decomposition during the electrochemical switching upon its formation. The minute amount of delaminates recovered from the continuous redox cycles precludes relative quantification of the isolated degradation products from the NMR spectrum. The exact mechanism, whether it be delamination followed by hydrolysis or in situ hydrolysis then delamination, cannot be equivocally assigned from the product analyses. Nonetheless, the data confirm the decrease in electrochromic performance arises from the ultimate degradation of the film.

**Figure 9:**
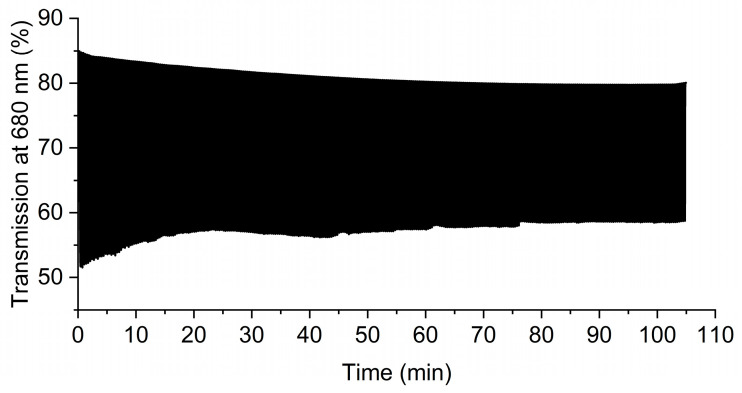
Change in transmission percent for the polymer in propylene carbonate (0.1 M TBAPF_6_) monitored at 680 nm when cycled between 1.2 and −0.1 V at 10 s intervals of applied potential.

In light of the identified performance degradation pathway of the film, a means to extend its electrochromic performance would be to minimize the degradations/hydrolysis of the film. The integrity of the azomethine bonds, and hence its electrochromism, could be potentially preserved by decreasing the time of applied potential. This would minimize the amount of degradation products formed during the anodic cycle, resulting in consistent transmission differences with repeated redox cycles. The film was subjected to shorter time intervals (10 s) of applied potential in propylene carbonate (0.1 M TBAPF_6_) to confirm the interplay of performance with the period of applied potential. The potential was switched between +1.2 V and −0.1 V and the transmittance at 680 nm was measured as a function of time. The electrochemical and spectroelectrochemical behavior of the polymer in the propylene carbonate electrolyte was consistent with the performance that was observed in dichloromethane (Figures S7 and S8). The stability of the polymer was improved at shorter switching cycles as per Figure 9 as expected. Indeed, the film could be switched between its oxidized and neutral states over 315 cycles. The initial transmittance difference between the blue (oxidized) and the red (neutral) states was 33.4%. The difference decreased by ~26% after ~20 oxidation/reduction cycles after which the optical difference remained constant. After 315 oxidation/reduction cycles the transmittance difference was 22%. This indicates that the exchange of the electrolyte and shortening of the interval of the applied potential improve the stability of the polymer layer.

**Figure 10:**
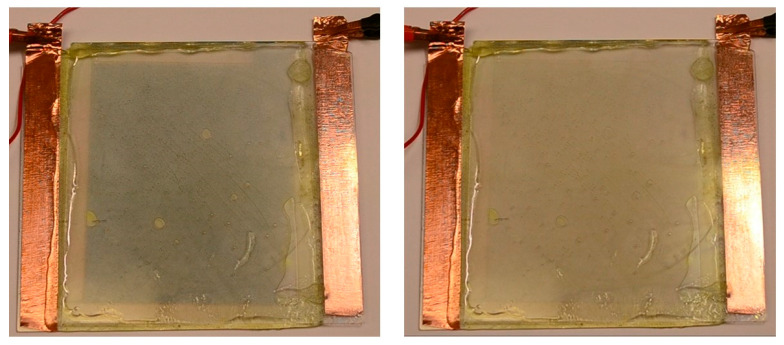
Pictures of the operating large-scale electrochromic device (10 cm × 10 cm) fabricated with the polyazomethine as the active layer at applied potentials of 2.6 (**left**) and −1.0 V (**right**).

The functioning small-area test device confirmed the suitability of the polyazomethine for use as an active layer in electrochromic devices. The advantage of forming the insoluble azomethine directly on the working electrode by spray coating is the size of the device that can be prepared is limited only by the size of the substrate. Another benefit of automating the coating process is that films of consistent thickness and surface coverage can be obtained. This is of importance because the quality (thickness and roughness) of the resulting polymer layer is governed by the quality of the monomer coating [11,14,39]. To demonstrate the scalability of the spray coating method, a device that was 16-fold larger in area than the test device was subsequently fabricated. The large-area device was prepared in the same manner by coating an equimolar concentration of the monomers **A** and **B** with an automatic sprayer directly on a large substrate (100 cm^2^). The same polymerization conditions were employed. The performance of the larger scale electrochromic device was consistent with its smaller test-size counterpart at applied potentials of 2.6 V and −1.0 V (Figure 10). The performance of the device at different time intervals is demonstrated in the Supplementary Video S1. It can be seen that both the coloration and the bleaching of the large area device occur within a few seconds. Such coloration kinetics meet the performance requirements for electrochromic applications such as smart windows.

## 4. Conclusions

Electrochromic layers could be prepared directly on a transparent electrode by a straightforward approach. Indeed, immobilized poly(triphenylamino azomethine) electrochromic layers could be prepared by coating the electrode with complementary diamino/dialdehyde monomers and subsequent annealing. The *spray-and-bake* coating process is also suitable for upscaling the preparation of working electrochromic devices. This opens the possibility of fabricating smart windows that have consistent electrochromic layers across their entire surface. Devices prepared by the *spray-and-bake* method had the benefit of fast bleaching/coloration times (<3 s). The performance decay was underpinned by the applied potential intervals with the performance linked to hydrolysis during extended cycles of applied potential. Device performance could be improved by reducing the cycling interval of applied potential. Extended switching performance of on-substrate prepared devices can ultimately be achieved by using multifunctional monomers. These will yield highly cross-linked electrochromic layers capable of meeting the multiple bleaching/colorations requirements of smart windows by limiting the hydrolysis degradation mechanism during device operation.

## Figures and Tables

**Table 1:** Reported electrochromic performance of polyazomethines that are structurally comparable to the on-substrate prepared polyazomethine.

Polyazomethine	Perceived Color Change	λ (nm) ^a^	Response Time (s)		Ref.
			T_c_	T_b_	
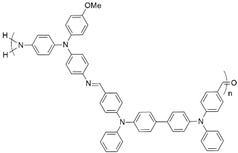	Yellow → red ↔ blue	680 nm	3.3	1.2	This work
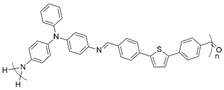	Yellow ↔ yellowish-green	428 nm	4.54	3.88	[66]
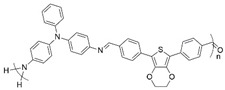	Yellow ↔ bluish-green	411 nm	2.23	1.90	[66]
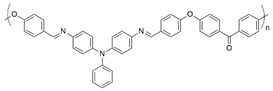	Yellow ↔ red	717 nm	5.17	4.01	[67]
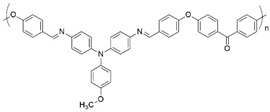	Yellow ↔ blue	725 nm	2.01	3.15	[67]
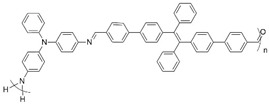	Orange ↔ light purple	905 nm	6.45	1.07	[68]

^a^ Corresponding wavelength of the spectroelectrochemical properties.

## Data Availability

Data is contained within the article or Supplementary Materials.

## Supplementary Materials

The following supporting information can be downloaded at: https://www.mdpi.com/article/10.3390/polym16172440/s1, Figure S1: Synthetic scheme for the preparation of monomers **A** and **B**; Figure S2: FT-IR spectra of **A** (black) **B** (red) as a thin film coating of equimolar **A** and **B** on ITO coated glass (green) and the resulting polyazomethine (blue); Figure S3: Cyclic voltammogram of the polyazomethine; Figure S4: Proposed oxidation/reduction mechanisms of the polyazomethine layer; Figure S5: Change in percent transmission measured at 545 nm for the test-size electrochromic device cycled between 2.3 V and −1 V at 30s switching intervals; Figure S6: Change in transmission percent for the test-size electrochromic device monitored at 680 nm when cycled between 2.6 and −1 V at 30 s intervals; Figure S7: Cyclic voltammogram of the polymerized azomethine on an ITO working electrode measured in propylene carbonate with TBAPF_6_ (0.1 M) as the supporting electrolyte; Figure S8: Absorbance spectra of the polymerized azomethine on an ITO working electrode measured in propylene at 0 (■), 0.4 (●), 0.5 (▲), 0.6 (▼), 0.7 (♦), 0.8 (◄), 0.9 (□), 1.0 (○), 1.1 (Δ), 1.2 (◊) and 1.3 V (□); Figure S9: ^1^H-NMR spectrum of **A** in CDCl_3_; Figure S10: ^13^C-NMR spectrum of **A** in CDCl_3_; Figure S11: ^1^H-NMR spectrum of **B** in CDCl_3_; Figure S12: ^13^C-NMR spectrum of **B** in CDCl_3_; Figure S13: ^1^H-NMR spectrum of **1** in CDCl_3_; Figure S14: ^13^C-NMR spectrum of **1** in CDCl_3_; Figure S15: ^1^H-NMR spectrum of **2** in CDCl_3_; Figure S16: ^13^C-NMR spectrum of **2** in CDCl_3_; Video S1: The operation of large-scale electrochromic device (10 cm × 10 cm) fabricated with the polyazomethine as the active layer.
