# Supplementary material for: On-Substrate Preparation of a Poly(triphenylamino azomethine) for Electrochromic Devices
Source: Polymers (Basel). 2024 Aug 28;16(17):2440. doi: 10.3390/polym16172440 (PMC11397927; doi:10.3390/polym16172440)
Supplement: Supplementary file 1 [file polymers-16-02440-s001.zip › supplementary information.pdf]

SUPPLEMENTARY INFORMATION

On-Substrate Preparation of a Poly(triphenylamino azomethine) for Electrochromic Devices

Heather L. Filiatrault,[1] Kacper Muras,[2] Monika Wałesa-Chorab,[1,2*] W. G. Skene[1,3]*

[1] *Laboratoire de caractérisation photophysique des matériaux conjugués*

Département de Chimie

Université de Montréal, CP 6128, succ. Centre-ville,

Montréal, Québec, Canada H3C 3J7

[2] *Faculty of Chemistry, Adam Mickiewicz University in Poznań, Uniwersytetu Poznańskiego 8, 61-614 Poznań, Poland*

[3] *Institut Courtois*

Université de Montréal, CP 6128, succ. Centre-ville,

Montréal, Québec, Canada H3C 3J7

Table of Contents

Figure S1. Synthetic scheme for the preparation of monomers **A** and **B**. 3
Figure S2. FT-IR spectra of **A** (black) **B** (red) as a thin film coating of equimolar **A** and **B** on ITO coated glass (green) and the resulting polyazomethine (blue). 3
Figure S3. Cyclic voltammogram of the polyazomethine. 4
Figure S4. Proposed oxidation/reduction mechanisms of the polyazomethine layer. 4
Figure S5. Change in percent transmission measured at 545 nm for the test-size electrochromic device cycled between 2.3 V and -1 V at 30s switching intervals. 5
Figure S6. Change in transmission percent for the test-size electrochromic device monitored at 680 nm when cycled between 2.6 and -1 V at 30 s intervals. 5
Figure S7. Cyclic voltammogram of the polymerized azomethine on an ITO working electrode measured in propylene carbonate with $TBAPF_6$ (0.1 M) as the supporting electrolyte. 6
Figure S8. Absorbance spectra of the polymerized azomethine on an ITO working electrode measured in propylene at 0 (■), 0.4 (●), 0.5 (▲), 0.6 (▼), 0.7 (♦), 0.8 (◄), 0.9 (□), 1.0 (○), 1.1 (Δ), 1.2 (◊) and 1.3 V (□). 6
Figure S9. ^{1}H-NMR spectrum of **A** in $CDCl_3$. 7
Figure S10. ^{13}C-NMR spectrum of **A** in $CDCl_3$. 7
Figure S11. ^{1}H-NMR spectrum of **B** in $CDCl_3$. 8
Figure S12. ^{13}C-NMR spectrum of **B** in $CDCl_3$. 8
Figure S13. ^{1}H-NMR spectrum of **1** in $CDCl_3$. 9
Figure S14. ^{13}C-NMR spectrum of **1** in $CDCl_3$. 9
Figure S15. ^{1}H-NMR spectrum of **2** in $CDCl_3$. 10
Figure S16. ^{13}C-NMR spectrum of **2** in $CDCl_3$. 10

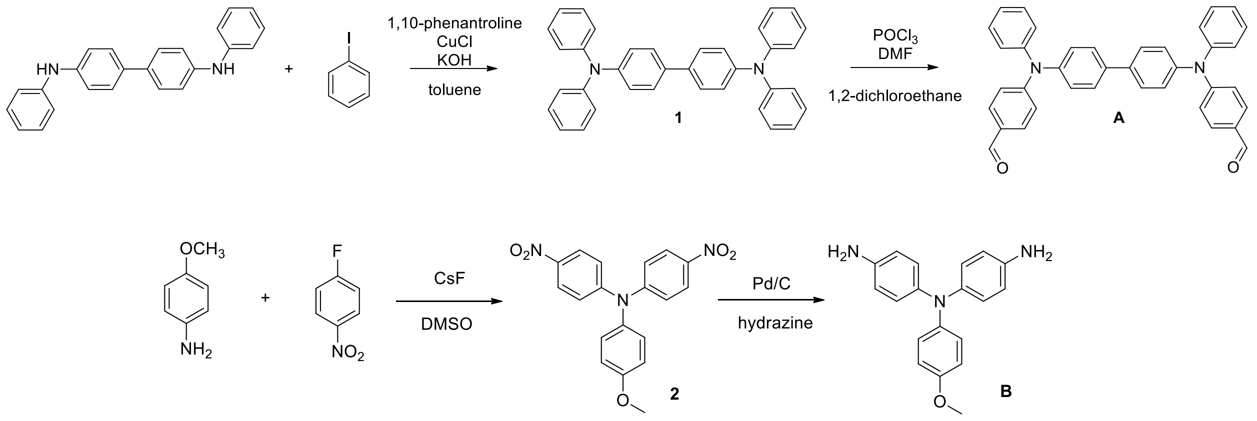

Figure S1. Synthetic scheme for the preparation of monomers **A** and **B**.

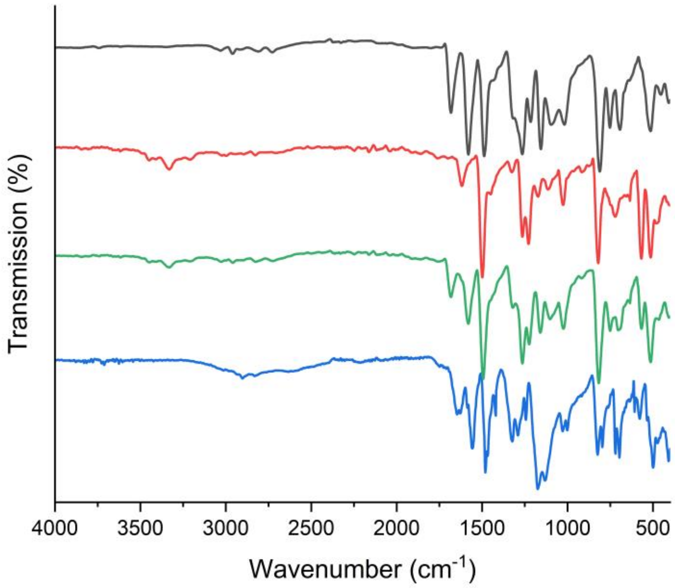

Figure S2. FT-IR spectra of **A** (black) **B** (red) as a thin film coating of equimolar **A** and **B** on ITO coated glass (green) and the resulting polyazomethine (blue).

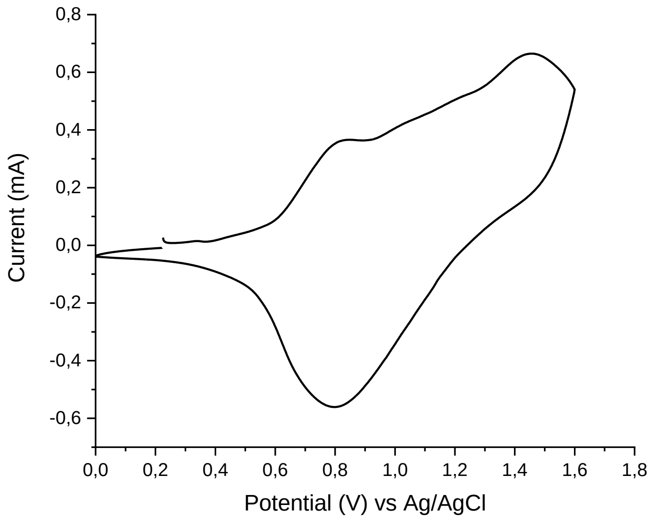

Figure S3. Cyclic voltammogram of the polyazomethine.

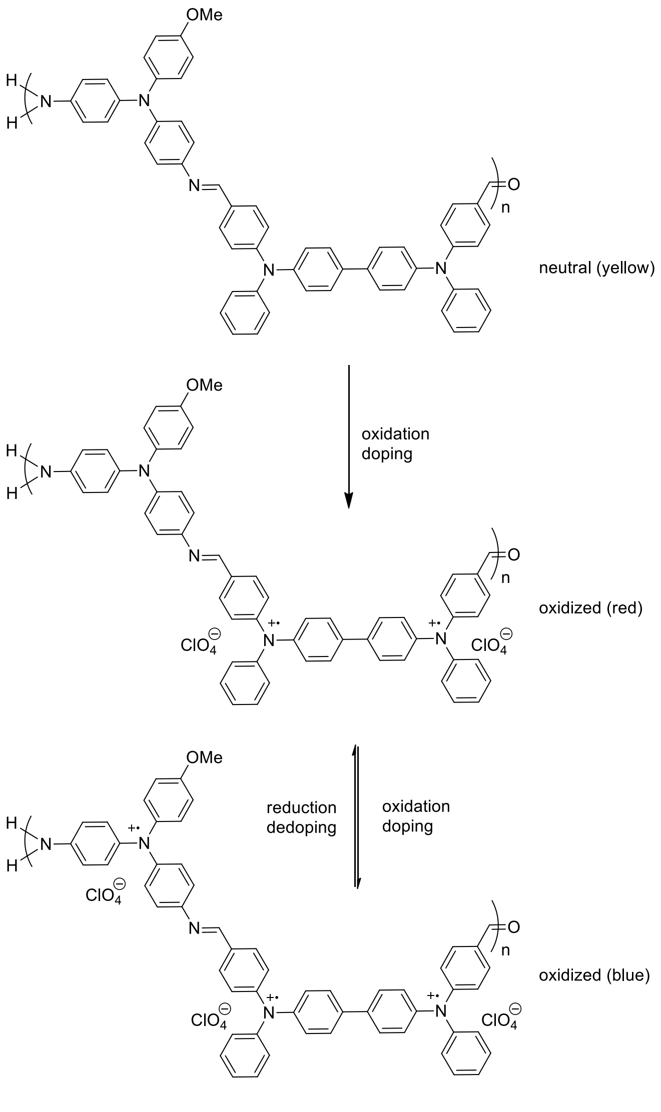

Figure S4. Proposed oxidation/reduction mechanisms of the polyazomethine layer.

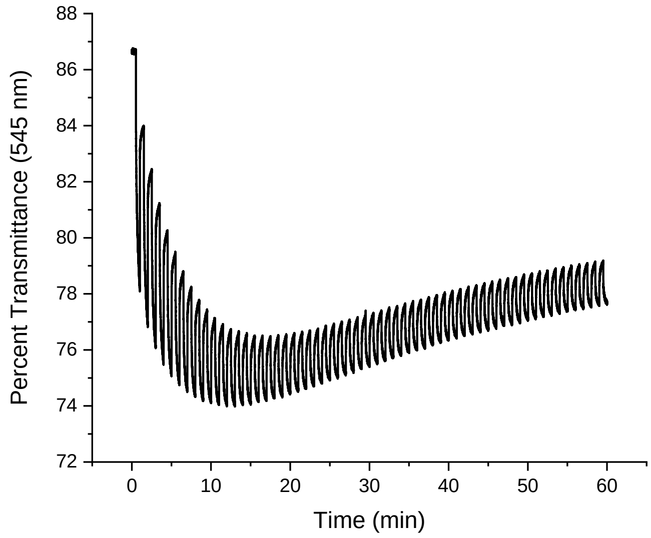

Figure S5. Change in percent transmission measured at 545 nm for the test-size electrochromic device cycled between 2.3 V and -1 V at 30s switching intervals.

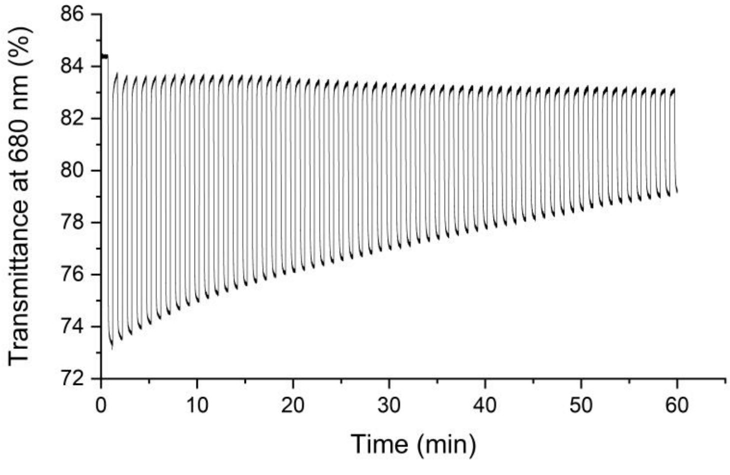

Figure S6. Change in transmission percent for the test-size electrochromic device monitored at 680 nm when cycled between 2.6 and -1 V at 30 s intervals.

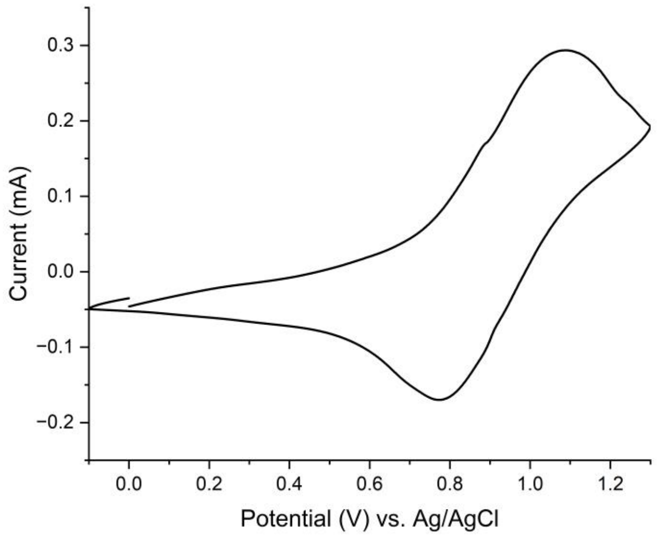

Figure S7. Cyclic voltammogram of the polymerized azomethine on an ITO working electrode measured in propylene carbonate with $TBAPF_6$ (0.1 M) as the supporting electrolyte.

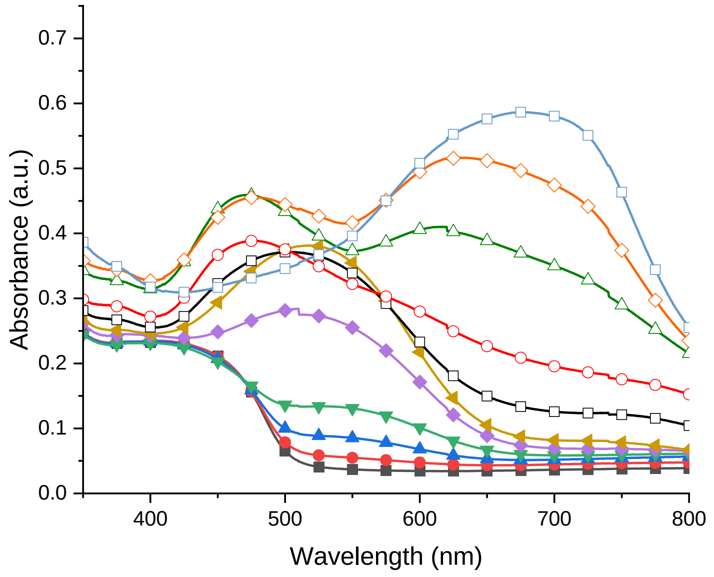

Figure S8. Absorbance spectra of the polymerized azomethine on an ITO working electrode measured in propylene at 0 (■), 0.4 (●), 0.5 (▲), 0.6 (▼), 0.7 (♦), 0.8 (◀), 0.9 (□), 1.0 (○), 1.1 (△), 1.2 (◊) and 1.3 V (□).

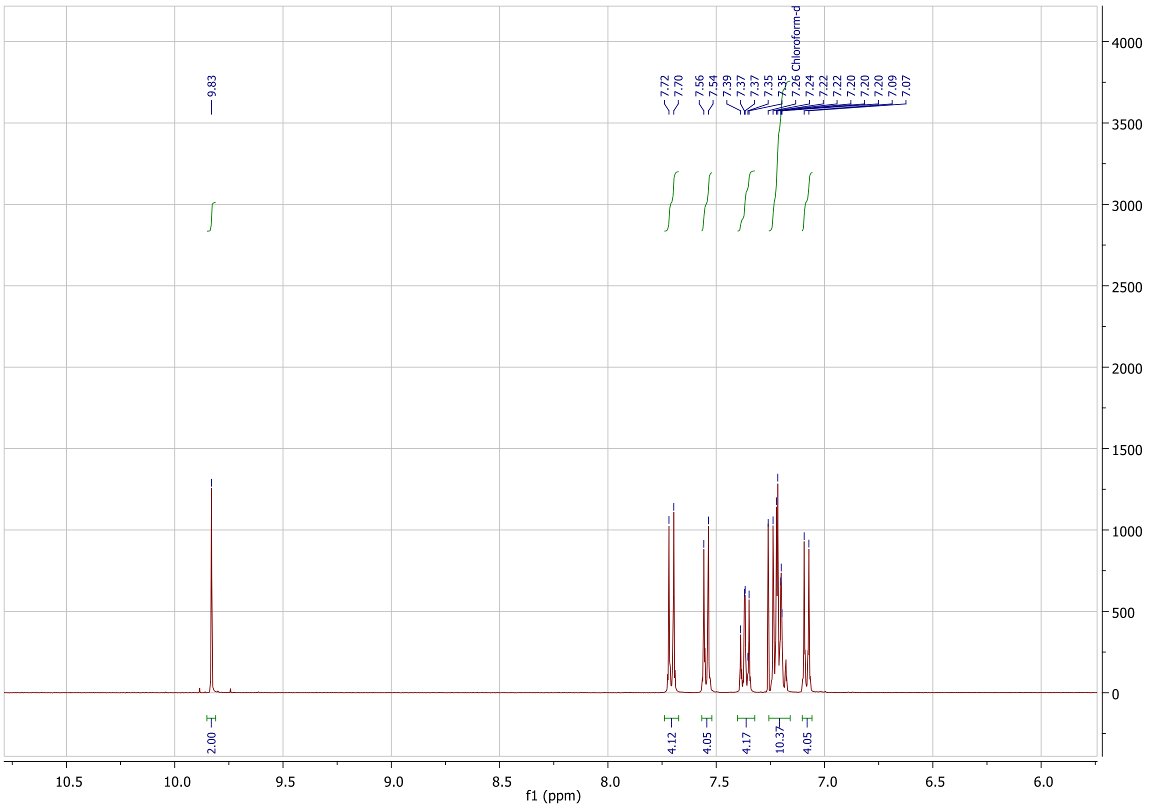

Figure S9. ^{1}H-NMR spectrum of **A** in $CDCl_3$.

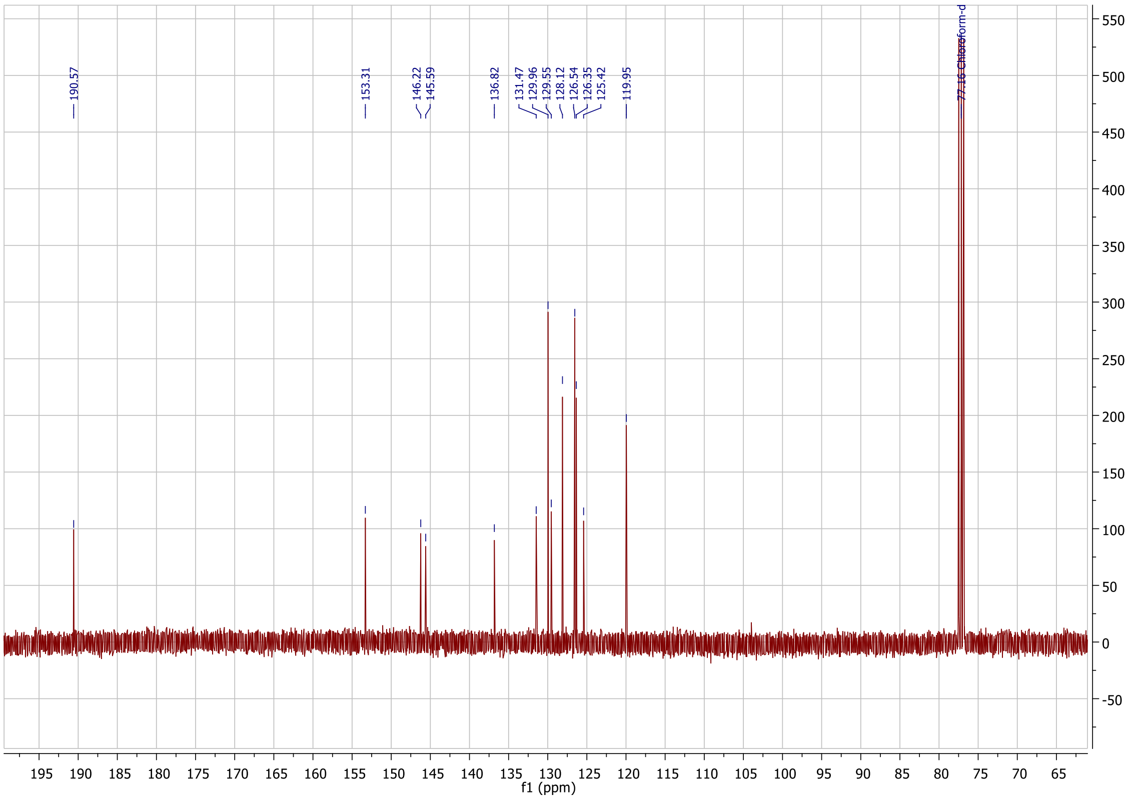

Figure S10. ^{13}C-NMR spectrum of **A** in $CDCl_3$.

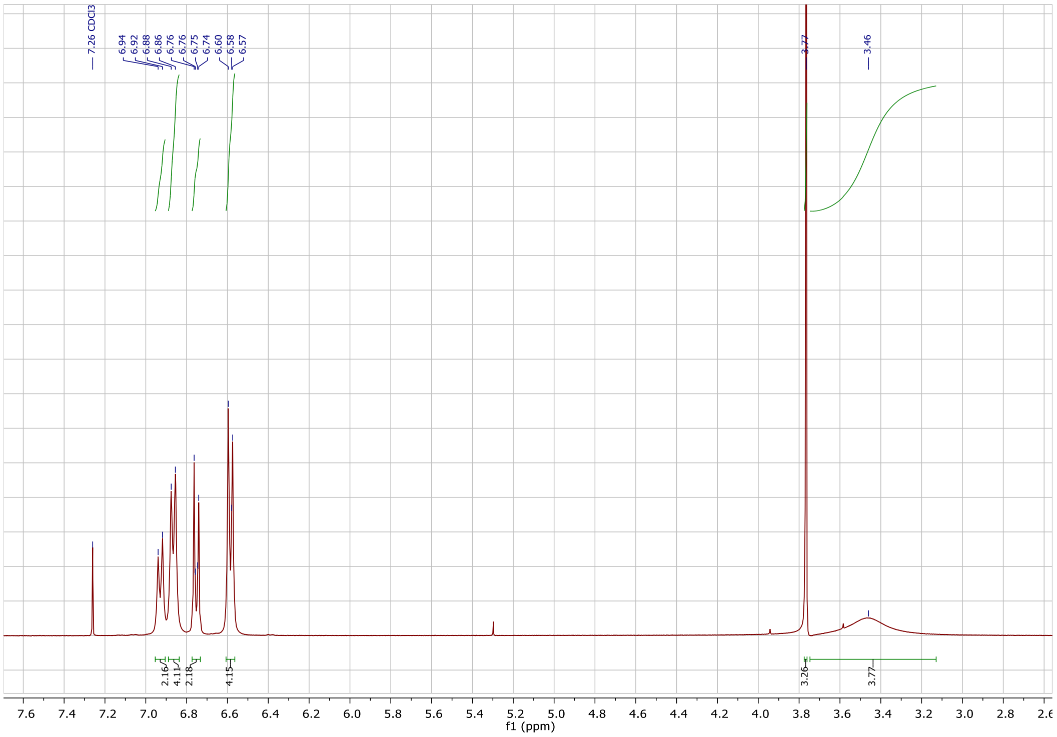

Figure S11. ^{1}H-NMR spectrum of **B** in $CDCl_3$.

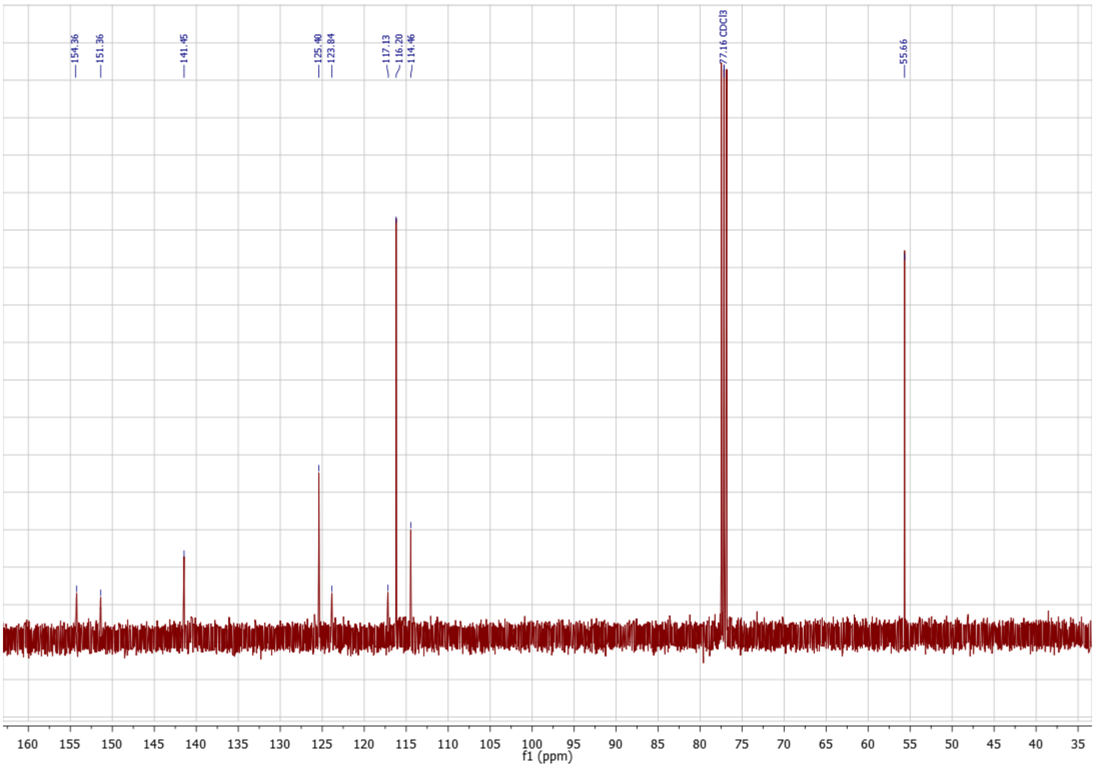

Figure S12. ^{13}C-NMR spectrum of **B** in $CDCl_3$.

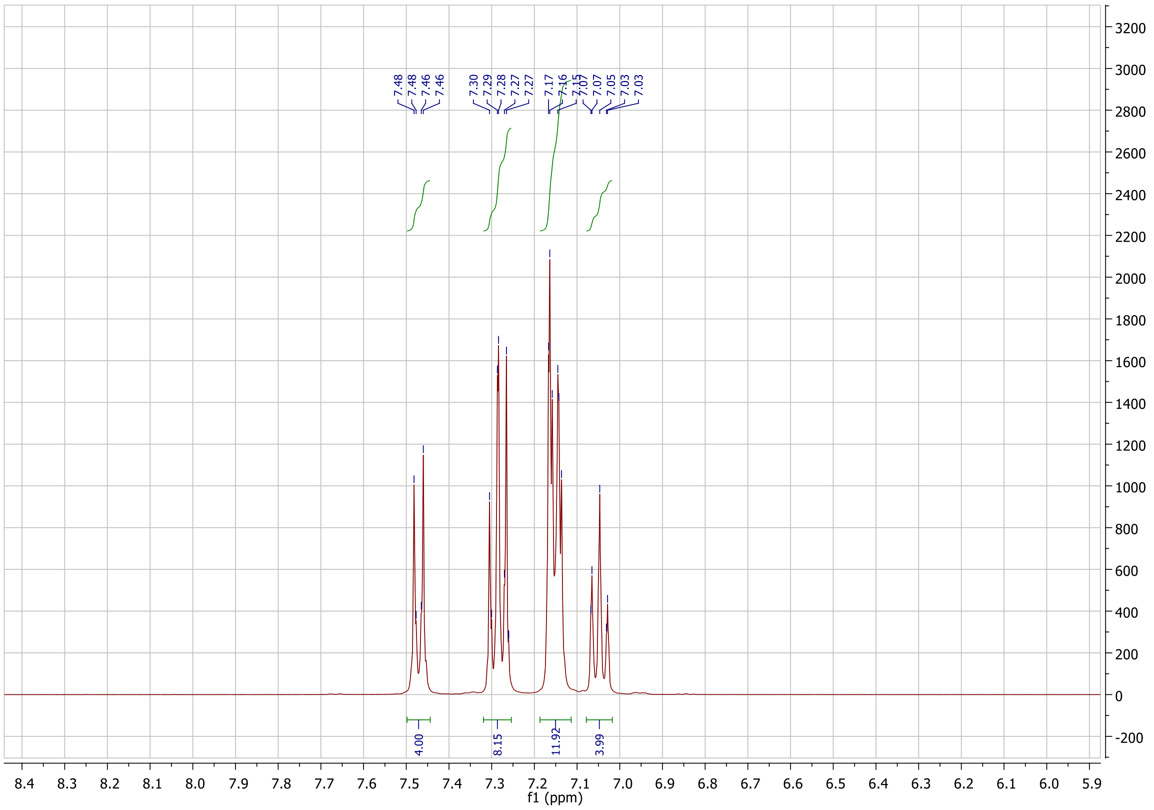

Figure S13. ^{1}H-NMR spectrum of **1** in $CDCl_3$.

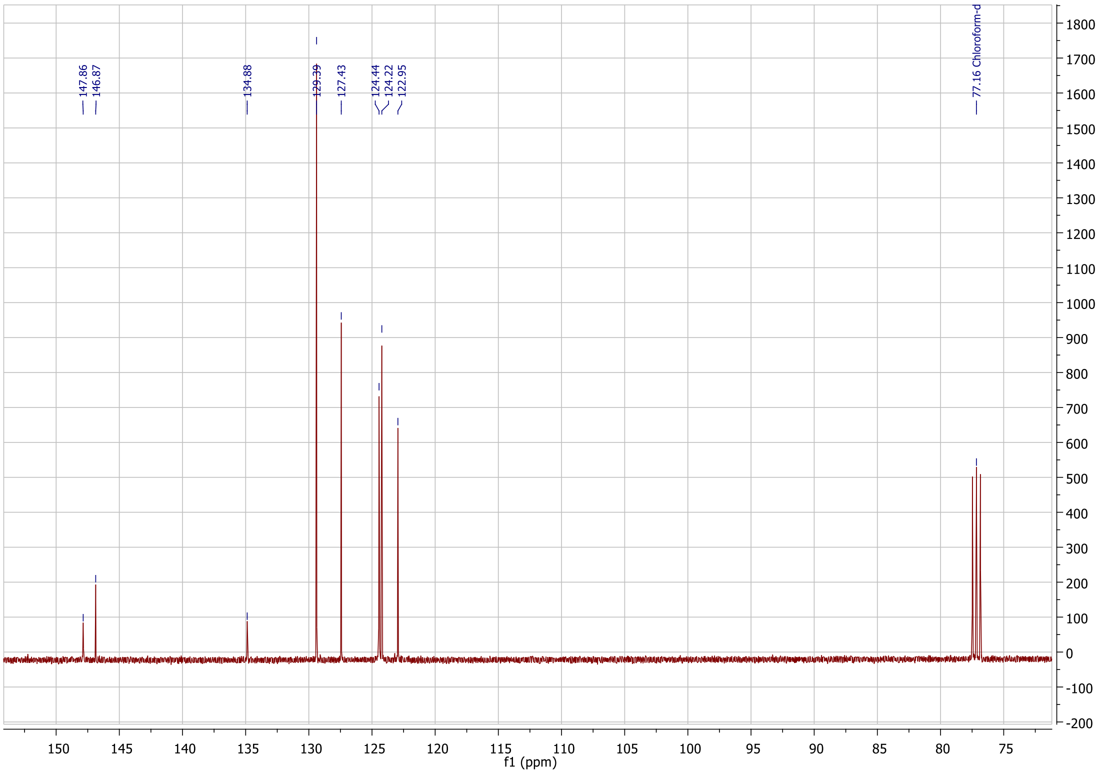

Figure S14. ^{13}C-NMR spectrum of **1** in $CDCl_3$.

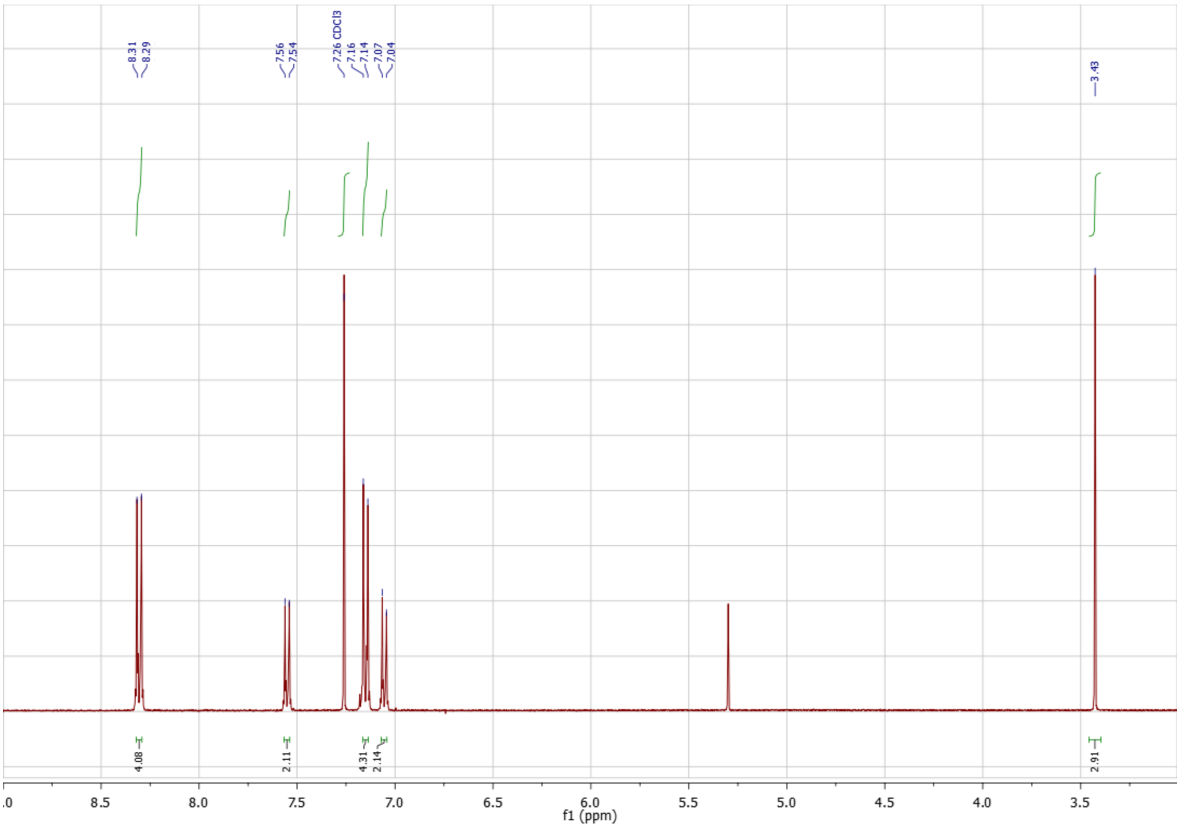

Figure S15. ^{1}H-NMR spectrum of **2** in $CDCl_3$.

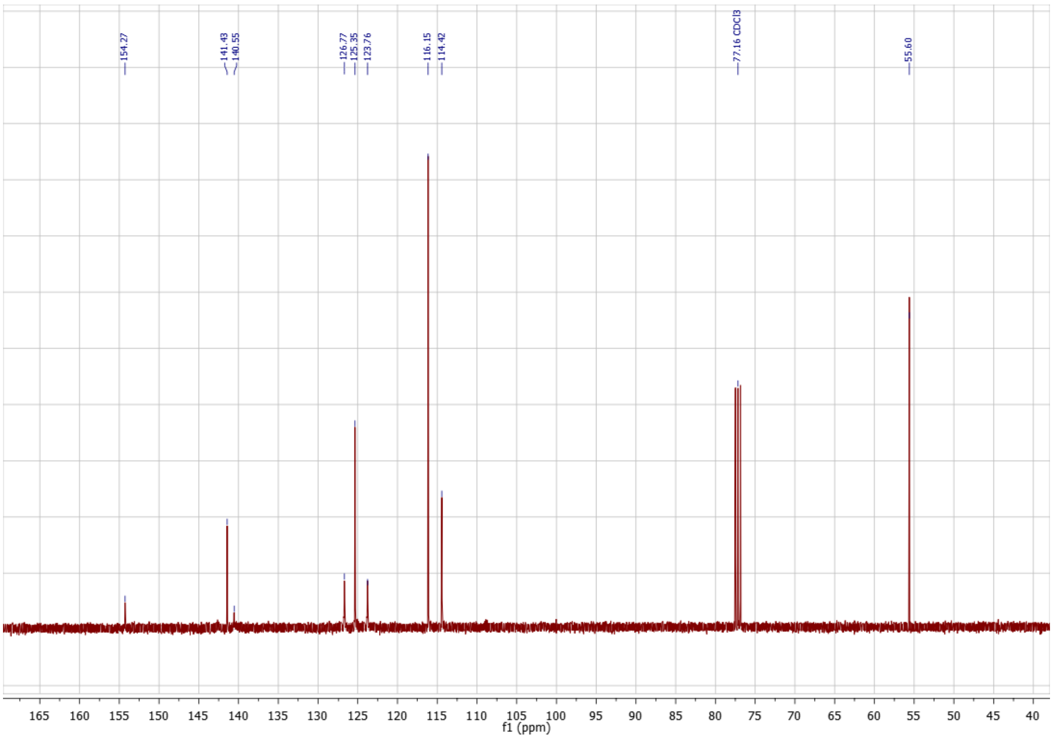

Figure S16. ^{13}C-NMR spectrum of **2** in $CDCl_3$.
